# Comorbidities of Chronic Urticaria: A glimpse into a complex relationship

**DOI:** 10.3389/falgy.2022.1008145

**Published:** 2022-11-17

**Authors:** Niki Papapostolou, Paraskevi Xepapadaki, Alexander Katoulis, Michael Makris

**Affiliations:** ^1^ Allergy Unit, 2nd Department of Dermatology and Venereology, Medical School, National and Kapodistrian University of Athens, Attikon University Hospital, Athens, Greece; ^2^ Allergy Department, 2nd Pediatric Clinic, National and Kapodistrian University of Athens, Athens, Greece

**Keywords:** chronic urticaria, comorbidities, chronic spontaneous urticaria, psychiatric disorders, atopic diseases, autoimmune diseases

## Abstract

Chronic Urticaria (CU) is a chronic inflammatory, predominantly mast cell-driven disease, characterized by the development of wheals and/or angioedema for more than 6 weeks. It affects approximately 1%–5% of the total population worldwide and imposes a substantial burden on health-related quality of life, significantly affecting patients' daily life. The economic impact on the health system is also not negligible, with an estimated cost per patient per year of approximately 2.000 $ in the United States. Although the underlying pathophysiology is not fully explored, autoimmune mechanisms have been proposed, including type I (“autoallergy” by means of autoantibodies to self-antigens) and type IIb (autoimmunity). Atopic, autoimmune, and psychiatric disorders are prevalent comorbidities in both children and adults with Chronic Spontaneous Urticaria (CSU). Although malignancies, cardiovascular diseases and other comorbidities have also been reported as associated diseases in patients with CSU, data remain scarce. It is still unknown whether the aforementioned comorbidities share common pathophysiological mechanisms with specific endotypes of CSU. The current review aims to overview current data on comorbidities of CU, and furthermore to comment on the potential linked pathways underlying these diseases.

## Graphical Abstract

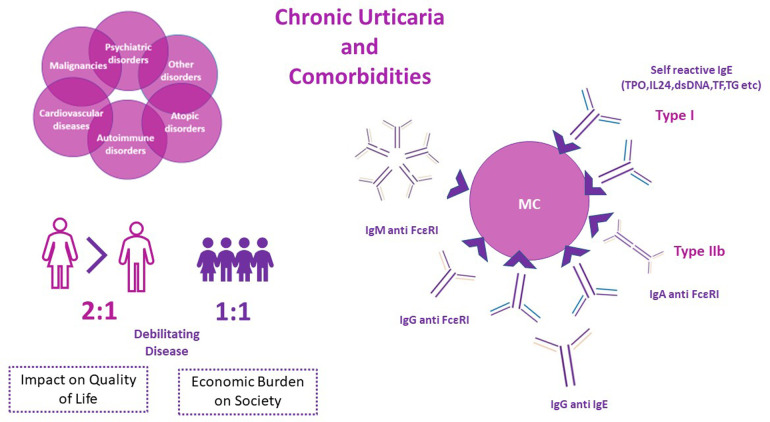

## Introduction

Chronic Urticaria (CU) is a predominantly mast cell-driven disease presenting with recurrent wheals, angioedema, or both for more than six consecutive weeks (1, 2). The disease is further classified into Chronic Inducible Urticaria (CIndU) and Chronic Spontaneous Urticaria (CSU), based on the presence or absence of specific causative triggers respectively (2), while 10%–30% of the patients with CU present both the spontaneous and inducible type (3).

CU is one of the most common skin disorders, with an estimated global prevalence ranging from 1% to 5% (4–6), both in children and adults, while data support an increasing prevalence worldwide, despite substantial regional disparities.(4) Females are slightly more affected compared to males (7, 8), with an increased point incidence of 0,18% vs. 0,11% and prevalence of 0,62%–1.3% vs. 0,37%–0.8% respectively (9). Such discrepancies are not present in the pediatric population (boys 1, 1% vs. girls 1, 0%) (4).

While CU affects all age groups, it is more frequent in patients aged 30–50 years (10), and thus influences mostly young and middle-aged women (11), compromising not only the quality of life but also work productivity and emotional well-being (12, 13). The socioeconomic burden is also substantial with an estimated cost per patient per year of 2,047$ in the United States and total direct and indirect costs accounting for 244$ million per year (14).

CSU is considered a chronic inflammatory skin disease and mast cells (MC) are undoubtedly the key effector cells, while various other cells and mediators are involved (15). The crucial role of basophils in CSU has recently been explored, revealing new aspects of CSU pathomechanisms (16). Blood basophil counts in patients with CSU inversely correlate with urticaria severity, and basopenia *per se* is linked with poor response to omalizumab treatment (17–19). Moreover, basophil infiltration has been detected in urticarial skin lesions, indicating a possible migration of these cells to the skin (20). Omalizumab administration has been associated with increased blood basophil counts and surface activation markers (21, 22). Based on this observation and omalizumab kinetics regarding rapid downregulation of Fc*ε*RI on the surface of basophils, Takimoto- Ito et al. hypothesized that activated basophils in CSU patients migrate to the skin. In contrast, inactive ones remain in the bloodstream. Upon omalizumab administration and urticaria resolution, levels of activated basophils increase in the blood, further highlighting basophils' role in CSU (16).

Although the underlying mechanisms of CSU remain largely unclear an autoimmune basis was first proposed in 1962 (23) and during the last decade two different endotypes have been described and classified as type I and Type IIb autoimmune mechanisms (24–27). In type I autoimmunity or “autoallergy”, activation of mast cells is driven by an IgE mediated reaction against an endogenous allergen (autoantigen) such as thyroid peroxidase (TPO), interleukin-24, double- stranded DNA, tissue factor, thyroglobulin etc (28–31). In type IIb, IgG autoantibodies, and to a less extent IgM and IgA autoantibodies, are directed against IgE or its high affinity receptor (Fc*ε*RI) resulting in activation of MCs (28, 32–35). The presence of MC activating autoantibodies can be identified by the autologous serum skin test (ASST), basophil tests (BTs) and immunoassays (32). Low total IgE levels and elevated IgG against TPO are present in type IIb autoimmune CU and are inversely correlated in patients belonging to this endotype (32) Coexistence of IgG and IgE autoantibodies against the same endogenous antigen has also been reported (36). Multiple other triggers can activate MCs resulting in different, yet unexplored, non-autoimmune endotypes of CU (37). Apart from high (Fc*ε*RI) and low affinity (Fc*ε*RII) IgE receptors in the surface of MCs, numerous other receptors are capable of activating MC, such as Mas-Related GPR family member X2 (MRGPRX2) for substance P, eosinophilic peroxidase and major basic protein, C5a receptor for anaphylatoxins, CRTh2 for Prostaglandin D2(PGD2), cKit for stem cell factor (SCF), cytokine receptors like IL-4R*α*, IL5R, and TSLP-R, Toll-Like Receptors (TLRs) for pathogen-associated molecular patterns (PAMPs) and damage-associated molecular patterns (DAMPs). Moreover, inhibitory receptors like Siglec-8 and CD200R exist on the surface of MCs (38–40). Endothelial cells and the coagulation system have also been implicated in CU pathogenesis (40), as well as the dysregulation of intracellular signals within mast cells and basophils (37, 41). Moreover, aggregation and stacking of highly lipophilic IgE molecules can result in crosslinking of Fc*ε*RI in the absence of antigen binding (42).

**Figure 1:**
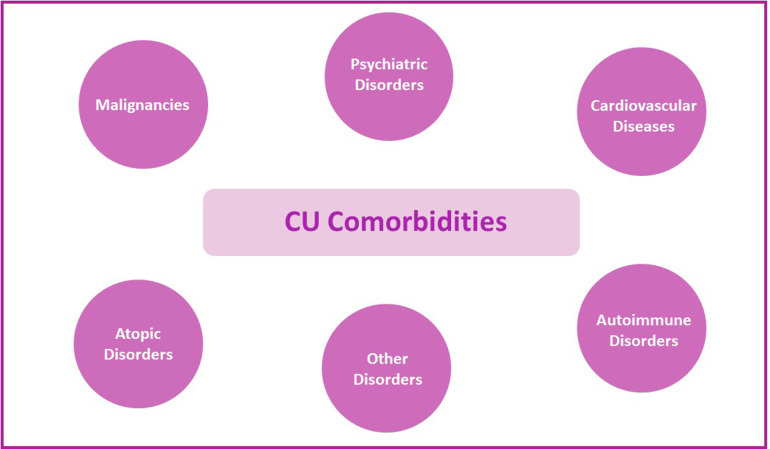
Chronic Urticaria frequently presents with various associated diseases (comorbidities). The robust link between atopic diseases, autoimmune diseases, psychiatric disorders and Chronic Urticaria is well established. A less clear relationship between malignancies, cardiovascular disorders of other comorbidities in patients with Chronic Urticaria exists.

Pruritus, pain and burning sensation of wheals and angioedema can result in anxiety, stress, sleeplessness, poor self-esteem, shyness, anger, and social isolation (43, 44). Furthermore, patients' quality of life is further compromised by the coexistence of CU with a broad spectrum of comorbidities, such as sleep disorders, anxiety, depression, other psychiatric disorders, autoimmune diseases, atopic diseases, cardiovascular disorders, and less frequently malignancies (9, 45–47) (Figure 1).

The above-mentioned data on CU pathophysiology and the underlying immune pathways has raised the interest for a more holistic approach of CU; to this end, both epidemiological data and possible common pathophysiological mechanisms linked to CU comorbidities are of major interest. In the present review we aim to overview data on the complex interplay between CSU and associated comorbidities, apart from CIndUs, and comment on their potential relationships in terms of underlying mechanisms.

## CSU and autoimmunity

CSU as an autoimmune-autoreactive skin disorder *per se*, often coexists with a variety of other autoimmune diseases (37). Overall, approximately 30% of CSU patients present with at least one autoimmune disorder, while 2% may have two or more autoimmune disorders, with Hashimoto's disease and vitiligo presenting more frequently as co-existent diseases (48).

Thyroid diseases have been reported as the most prevalent autoimmune diseases in up to 50% of CSU patients, depending on the study population (32, 49–51). Other autoimmune diseases as vitiligo (prevalence >3%), pernicious anaemia (>5%), rheumatoid arthritis (>1%), psoriasis (>1%), celiac disease (>1%) and insulin-dependent diabetes mellitus (>1%) have also been reported in CSU patients (50, 52, 53). From another perspective, the prevalence of CSU is higher, in patients suffering from Systemic Lupus Erythematosus (SLE), rheumatoid arthritis, autoimmune thyroid diseases, and celiac disease compared to the general population (49, 52).

It has been recently acknowledged that the type IIb autoimmune CSU, as assessed by positive ASST, BHRA and/or BAT and identification of specific IgG antibodies against Fc*ε*RI/IgE, is highly related with other autoimmune diseases (48).

### Thyroid diseases

IgG autoantibodies against thyroid peroxidase (TPO) have been identified in up to 50% of CSU patients, with 5-to-7-fold increased risk of presenting anti-TPO antibodies in CSU patients compared to controls, while increased levels of IgE antibodies against TPO have also been detected in those subjects (49, 54). Thyroid dysfunction disorders, such as hypothyroidism and Hashimoto's thyroiditis, are also reported more significantly in CSU adult patients than healthy controls (49).

First, Rumbyrt et al. suggested that the inflammation in the thyroid gland can lead to a generalized inflammatory response with a subsequent complement activation along with activation of mast cells, mainly through anaphylatoxins receptors (55). Moreover, the recognition of IgE antibodies against TPO as a cause of Type I autoimmune CSU has further enhanced the link between thyroid dysfunction and CSU (25, 56). In line, although a causative role of IgG antithyroid autoantibodies on the occurrence of CSU has not been demonstrated (57–59), IgE antithyroid autoantibodies have been implicated in the formation of immune complexes, and activation of complement system, potentially facilitating activation of MCs and subsequent clinical expression of CSU (49).

Although conflicting evidence exists, especially in euthyroid patients with CSU, data support the efficacy of levothyroxine or other thyroid drugs on CSU morbidity, potentially by reducing inflammatory thyroid pathways mediating mast cell activation (49).

### Other autoimmune diseases

In a large registry-study from Denmark including more than 12.000 CU patients, rheumatoid arthritis was reported as the most prevalent autoimmune comorbidity (1.7%), while thyroiditis (0.3%), vitiligo (0.1%) and Systemic Lupus Erythematosus (SLE) (0.3%) were also identified, although to a lower extend. Of note, it cannot be excluded that the high prevalence of RA might be attributed to the high prevalence of the disease *per se*, in relation to the other autoimmune diseases. At the day of the diagnosis, rates of vitiligo and SLE were significantly higher than in the control group (OR = 5.43 1.78–15.35 and OR = 4.72 2.36–7.4 respectively). During the follow up, an increased risk for RA occurrence was observed [Hazard Ratio = 1.8 (1.4–2.3)] (51). This could potentially be attributed to the systemic inflammation facilitated by MCs, while the role of MC's activating autoantibodies might be more relevant in CU patients with autoimmune thyroid diseases, vitiligo and SLE (51).

Additionally, it is well known that Urticarial rash is common in patients with Systemic Lupus Erythematosus (SLE), ranging from 0.4%–27.5% in adults and in 4.5%–12% in children as shown in the meta-analysis by Kolkhir et al. Data on the vice versa relationship is scarce. It has been proposed that the underlying pathogenetic mechanism associating both diseases might include the activated complement and coagulation system, linking inflammation and autoimmunity (60).

### Autoimmune diseases in paediatric population

The prevalence of autoimmune diseases in children with CU is diverse, ranging from 0%–16% (61, 62). A prospective study in Canada, evaluating the prevalence of autoimmune diseases in children with CSU, demonstrated an increased prevalence of autoimmune diseases, such as hypothyroidism, lupus, juvenile rheumatoid arthritis, and type I diabetes compared to the general paediatric population (2.10% vs.0.13%, 0.52% vs.0.005%, 1.05% vs. 0.053% and 1.57 vs. 0.19% respectively) (63). Nevertheless, the overall prevalence of autoimmune diseases in children with CSU was relatively low (<5%), thus evaluation for autoimmune diseases is proposed only when a suggestive clinical history and/or laboratory findings are present (63). Moreover, autoimmune hypothyroidism was observed in older children with CSU and with increased CD63 levels, a well-established marker of IgG-mediated autoimmunity, potentially attributed to the impact of epigenetic changes, due to environmental factors, on the development of inflammation and autoimmunity with increasing age (63).

In respect to the prevalence of atopic diseases in children with CSU, studies have shown an increased occurrence compared to autoimmune diseases while in adults respective rates are either similar or even lower (49, 51, 63, 64). Moreover, in agreement with recent finding linking autoimmune type IIb endotype with higher prevalence of other coexisting autoimmune diseases in adults, elevated levels of CD63, may propose such a relationship in children as well (48, 63, 65).

A systematic review reported that positive ASST, identifiable antinuclear antibodies (ANA) and thyroid biological abnormalities were present in 36.8%, 6.4% and 10.4% of children <12 years with CSU respectively (66), supporting further the presence of a type IIb autoimmune endotype in children. The lower rates of thyroid function abnormalities are in line with the observation that autoimmune mechanisms are evolving and may manifest several years after the initial diagnosis (66). However, whether children with positive ASST and ANA need to be screened for autoimmune diseases is a matter of debate (67, 68).

### The importance of identifying autoimmune comorbidities in patients with CU

Specific endotypes of CSU are linked to comorbid autoimmune diseases, and thus early diagnosis and therapeutic intervention of associated diseases may be beneficial in the multidisciplinary therapeutic approach as suggested by EAACI/GA^2^LEN/EuroGuiDerm/APAAACI Guidelines (2, 48). In the era of precision medicine, knowledge of a patient's profile, shaped not only by CU *per se* but also by the various coexisting diseases, may lead to targeted, personalized interventions (69, 70). As new therapeutic options are developing, identifying the presence of comorbid autoimmune diseases is of importance, since they can interfere with CSU activity, duration, natural course, and response to treatment (69, 71). Thus, in the updated CU 2022 guidelines the measurement of IgG anti-TPO and total IgE in all CSU patients is strongly supported to identify autoimmune thyroiditis and to untangle the underlying endotype (2, 32).

## CU and atopic diseases

Atopic diseases have been commonly reported in CU patients. The results from the Scandinavian arm of the AWARE study, showed that atopic diseases are the most frequent comorbidities in a cohort of 158 adult patients with CU. In specific, asthma was reported in 19.6% of the patients, allergic rhinitis in 16.5%, atopic dermatitis in 6.3% and food allergy in 8.2% (11). Higher rates of sensitization -approximately 40%- to at least one inhalant or food allergen have been reported by Zuberbier et al. in a general German population with CU (72), while allergic rhinitis and asthma were among the five most common comorbidities among CU patients in a large Korean study (73). In agreement, Ghazanfar et al. found that atopic diseases like rhinoconjuctivitis and atopic dermatitis are overrepresented among CU patients with an increased risk of developing atopic diseases following CU diagnosis (HR = 3.09, CI 2.0–4.8 for atopic dermatitis and HR = 1.4, 0.75–2.55 for rhinoconjuctivitis) (51).

With regards to the pediatric population, a personal history of atopic dermatitis in children has identified as a risk factor for subsequent CSU development, (OR 2.92, 95% CI 1.64–5.18, *p* < 0,001) in a pediatric population (74). In addition, in a recent systematic review evaluating comorbidities and interventions in children younger than 12 years with CSU, including 522 patients with CU (or CSU), atopic diseases were found in 28.1% of the population with a reported prevalence of 15.4% for asthma, 13.8% for allergic rhinitis and 9.4% for atopic dermatitis respectively (66). In agreement, Lachover-Roth et al. in a retrospective study of 250 children with CSU showed that atopic diseases were significantly more prevalent in children with CSU than in the general paediatric population, with one out of three children suffering an atopic comorbidity (17.2% atopic dermatitis, 16% allergic rhinitis, 13.2% asthma and 3.2% food allergy) (75). Allergic sensitization, as assessed by total IgE has been identified in almost 30% of children with CU, irrespective of relevant clinical symptoms (76). Moreover, 24 out of 77 children with CU were described as atopic with presence of allergen specific-IgE to at least one allergen. Importantly, total levels of IgE were positively associated with disease duration. (r = 0.262, *p* = 0.021) (77). In CU adults, high IgE levels correlated with disease severity and duration, but not the clinical course of the disease (64, 78).

Despite the robust epidemiologic association between atopic diseases and CU, both in adults and children, no causal relationship has been established so far, thus therapeutic interventions for allergy-associated symptoms have no effect on the natural course or severity of CSU and vice versa (75, 79). Nevertheless, a TH2 endotype in CSU patients, especially children, with atopic diseases along with high IgE levels, which in turn are associated with type I autoimmunity or “autoallergy” and IgE autoantibodies detected in CSU patients, has been suggested (26, 34, 42, 75).

## CU and psychiatric disorders

Psychiatric and mental disorders are quite frequently reported among CU patients, in the literature (80–83). A recent systematic review and meta-analysis reported that almost one out of three CU patients have at least one underlying psychiatric disorder (84). Sleep-wake disorders, followed by anxiety and mood disorders, including depression are frequently identified (pooled prevalence 36.7%, 30.6% and 29.4% respectively). Trauma and stressor related disorders, somatic symptom and related disorders, obsessive- compulsive and related disorders and substance-related and addictive disorders were also reported. Regarding CU severity, duration, and mental functioning, no association has been demonstrated. Konstantinou et al. conclude that none of the studies included in the systematic review clearly stated whether psychiatric disorders pre-existed or follows CU diagnosis (84).

Data from the Danish National Patient Registry (*n* = 12.185 CU patients) found that CU patients were at increased risk of presenting depression, while a marginally increased risk for presenting psychosis was observed over time [HR adjusted = 1.38 (0.99–1.93) in CU patients] (51). Affective disorders (27.0%) were frequently in adults with CU in a cross-sectional study in Germany; of interest, in pediatric CU patients somatoform disorders were the most frequently reported comorbidities (7.7%), following rhinitis (24.7%) and asthma (20.2%) (9). Recently, Lachover-Roth et al. found a prevalence of 2.8% with respect to psychiatric disorders in a retrospective study of children with CSU (*n* = 380); depression, anxiety, bipolar disorders, and schizophrenia were identified (75).

Anxiety disorders are also prevalent in CSU patients compared to healthy controls (9.6% vs. 5.7%, *p* < 0.001), with a strongest association observed between anxiety, younger and higher socioeconomic status subjects (85). Moreover, anxiety can negatively correlated with social functioning (86).

Both anxiety and depression were negatively correlated with Quality of Life assessed by Chronic Urticaria Quality of Life Questionnaires (CU-QoL) (87).

Although a number of studies reports increased frequencies of depression and anxiety among CU patients (48,1% and 38% respectively) other reports show lower levels (11); discrepancies are potentially attributed to selection bias, heterogenous population and diagnostic criteria regarding diagnosis of psychiatric disorders (11).

Suicidal ideation is also reported in patients with CU (84). Picardi et colleagues (88) reported a 18.8% prevalence of suicidal ideation in CU patients, while Mehta et al. (89) and Sorour et al.(90) reported a 12% and 19.9% prevalence respectively.

The underlying pathogenetic mechanisms are unclear, although a potential interplay between the immune and central nervous system has been reported (91). A “brain-skin connection” may contribute to inflammatory skin diseases like CU, with stress causing aggravation of urticaria (92, 93). Moreover, a causal relationship between stress and inflammatory disorders, including CU, has been reported (94, 95). It has been postulated that chronic inflammation can dysregulate the immune and the central nervous system, resulting in mental disorders (96). The role of substance P, through neurogenic inflammation in acute stress has been described (97). Substance P is produced by a variety of inflammatory cells and is implicated in the release of histamine and serotonin from mast cells (98). In accordance, in a study evaluating patients with CSU and depression levels of Substance P were higher in CSU with depression than those without, but no dissimilarity was observed between CSU and healthy controls (99).

As CU has a debilitating effect on quality of life and productivity, data are inconclusive on whether psychiatric disorders affect or are affected by CU (84). Albeit case series have reported that pharmacological interventions with anti-depressants and anti- anxiety drugs may have a beneficial impact on CU (100, 101).

It is advised that CU patients be evaluated for phycological disorders and be treated accordingly.

## CU and malignancies

The association between CU and malignancies remains controversial (37). The first implication of a causal relationship between CU and cancer was described in 1942, when the removal of a rectal carcinoma in a 70-year-old male was associated with CU remission (102). Since then anecdotal cases of urticaria linked to malignancies have been reported in the literature (103).

Neoplasms have been reported to promote both chronic spontaneous and inducible urticaria in a systematic review, suggesting a linkage. The most frequently reported cancers in CSU patients are carcinomas (68%) with 24% of all cases being papillary carcinomas of the thyroid gland (103). In agreement, Napolitano et al., in a retrospective population-based study of 1,493 patients with CU, reported that CU was associated with cancer in 0,007% of the population, while CSU in those patients is (a) antihistamine resistant, (b) resolves after chemotherapy, or tumor removal, (c) can reoccur upon cancer relapse and (d) presents 2 to 8 months before malignancy diagnosis (103, 104). In accordance, a large registry study from Taiwan reported an increased risk of cancer in patients with CU (standardized incidence ratio 2.2; 95% CI 2.0–2.3). The risk was even higher for hematologic malignant tumors (SIR = 4.1, 95% CI, 3.1–5.4) and non-Hodgkin lymphomas (SIR = 4.4,95% CI, 3.0–6.1) (105). Moreover, two additional cases of urticaria remission after colorectal cancer removal are also reported in the literature, suggesting that urticarial lessons may manifest as a paraneoplastic phenomenon (106, 107). The incidence rates of CSU were statistically significantly higher for neoplasms (adjusted HR 1.14, 95% CI 1.02–1.27) in a population-based study in Italy (108). Non hematological neoplasms were among the most common comorbidities in a large Korean population-based study with the likelihood of occurrence 1.37 higher than in patients without CU. Stomach, thyroid and liver cancer were the most common neoplasms in CU patients while thyroid, liver and prostate in the CSU subgroup (73). In contrast, data from a Swedish registry showed no association between cancer and CU (109).

As urticaria and cancer are common diseases in the general population, they can incidentally coexist, although the immediate CU resolution following cancer remission and the reoccurrence upon relapse suggests causality (104). Neoplasms may induce immune dysregulation and activate coagulation and complement system, while the release of tumor-derived antigens detected by IgE can cause cross-linking of high-affinity IgE receptors in mast cells' surface, inducing degranulation (110–113).

Despite the reported cases in the literature, the overall rate is quite low among CSU patients and hence, the international EAACI/GA^2^LEN/ EuroGuiDerm /APAAACI guidelines suggest not to routinely screen for malignancies as potential underlying causes of CU (2, 114).

A careful clinical examination and history are essential for this rare relationship to be exposed in a cost -effective way.

## CU and hypertension, hyperlipidemia, metabolic syndromes, and cardiovascular disorders

The relationship between CU and cardiovascular diseases is unclear. A retrospective population-based cohort study in Denmark found no association between CU and cardiovascular diseases (115). On the contrary, a prospective study showed that systemic hypertension was associated with urticaria persistence (hazard ratio, 0.71; 95% CI 0.53–0.95; *p* = 0.02) (110), while hypertensive and lipoprotein metabolic disorders were among the more frequent reported comorbidities (43.5% and 32.1% of CU adult population respectively) in a recently published cross-sectional German study (9), and in a Swedish registry based-study (12% and 17% respectively) (116).

Metabolic syndrome was reported in 29.8% of patients with CU compared to 17.8% in a matched control group (*p* = 0,001) in a Korean cohort study and was independently correlated with uncontrolled urticaria, as assessed by total urticaria activity score. Larger waist-circumference, as a marker of obesity, was more prevalent in subjects with CU, and significantly associated with IgE, Eosinophilic Cationic Protein (ECP) and Tumor Necrosis Factor-a (TNF-a) levels (117), while a postive association between CU and obesity was shown in a large population-based Italian study (adjusted HR 1.40,95% CI 1.17–1.67) (108). Moreover, hyperlipidemia has been identified as a risk factor for CU development (OR 1.97 95% CI: 1.85–2.09) (118).

The Scandinavian arm of the AWARE study also reported a prevalence of obesity and hypertension at 7% and 1.9%, respectively, among an adult CU population with half of the patients being overweight (BMI > 25) (11).

Similarly, a pediatric cohort with CU from Spain, Italy, Germany, France, and the UK manifested significantly higher BMI compared to the control group (119).

CU is a chronic inflammatory disease presenting with low grade systemic inflammation (37). Hence, although an increased ratio of cardiovascular diseases derived from atherosclerosis could be partially explained by the inflammation stage in CU patients, data by Egeberg et al. report otherwise (115). The relatively short duration of CU may not be sufficient to increase the risk of presenting cardiovascular diseases (8, 120). However, alterations in lipid metabolism and co-occurrence of obesity can result in immune system dysregulation and presentation of autoimmune diseases (121, 122), with a subsequent activation of mast cells resulting in CU clinical presentation. Nevertheless, this hypothesis is far from well-established and further studies are needed to unravel the potential relationship between urticaria, hyperlipidemia, obesity, and cardiovascular diseases.

## CU and other comorbidities

Although less common, a variety of other associated diseases have been reported in patients with CU.

Osteoporosis and diabetes mellitus were found in 2.9% and 2.3% of 12.185 CU patients respectively (51). It is speculated that corticosteroid use plays a significant role as, despite current guidelines recommending against their use, they are still prescribed by physicians (2, 123, 124). The same study reported increased risk of having or achieving mastocytosis and anaphylaxis in the CSU group. However, the adjusted HR decreased when the diagnosis of these diseases within the first year were excluded, supporting a possible misdiagnosis before patients were referred to specialized centres (51). Drug allergy has also been identified to co-occur with CU with a likelihood of 4.68 times higher than in patients without CU (73).

Inflammatory diseases were the most prevalent comorbidities identified in a population-based study in Taiwan, with peptic ulcer (4.83%), hepatitis B or C (1.64%) and periodontitis (2.82%) presenting more frequently. In patients with persist CU, an increasing prevalence of inflammatory diseases was observed, indicating a possible link between inflammation and endurance of CU (125).

Back pain, acute upper respiratory infections, non-inflammatory disorders of the vagina, spondylosis, and gastritis were among other rare disorders detected by using the anonymized research database of the Institute for Applied Health Research in Berlin, including insured individuals with a diagnosis of CU (9).

Additionally, a systematic review assessing the relationship between CSU and Vitamin D levels revealed that Vitamin D levels in 12 out of 14 included studies were significantly lower in CSU patients compared to controls (34.3%–89.7% of CSU patients and 0%–68.9% in controls). No causal relationship was identified, although supplementation of vitamin D for 1–3 months might have a beneficial effect in CU course (126). In accordance, a systematic review assessing comorbidities in children with CU found low vitamin D levels in 69.1% of the children (66); however data from other studies are not confirmatory (64).

## Conclusion

CU presents with a wide range of associated comorbidities. Autoimmune, psychiatric, and atopic diseases are the most frequently reported associated diseases among CSU patients. Although the link between specific comorbidities and CU is solid, the potential interplay, regarding the nature of co-occurrence, is a recently explored era. The existing data cannot provide evidence in order to elucidate whether those diseases circling CU coexist independently with it or if a causal relationship, deriving from shared pathogenetic mechanisms, exists. Besides, if this is the case, a further unanswered question would be whether therapeutic interventions regarding comorbidities could interfere with CU's clinical course and vice versa. Therefore, prospective well-designed studies addressing the impact of various comorbidities on CU course and severity, as well as the impact of therapeutic interventions of comorbidities in both CU activity and natural course, are of urgent need. As we are marching into the era of personalized medicine, patients with CU should be recognized as a multimorbid group, and management should involve recognizing and treating any comorbid disorders in addition to urticaria management.
